# Supplementary material for: The causal relationship between the human gut microbiota and pyogenic arthritis: a Mendelian randomization study
Source: Front Cell Infect Microbiol. 2024 Nov 26;14:1452480. doi: 10.3389/fcimb.2024.1452480 (PMC11629706; doi:10.3389/fcimb.2024.1452480)
Supplement: Supplementary file 1 [file DataSheet1.zip › mendelian test/ebi-a-GCST90027693.csv_forest.pdf]

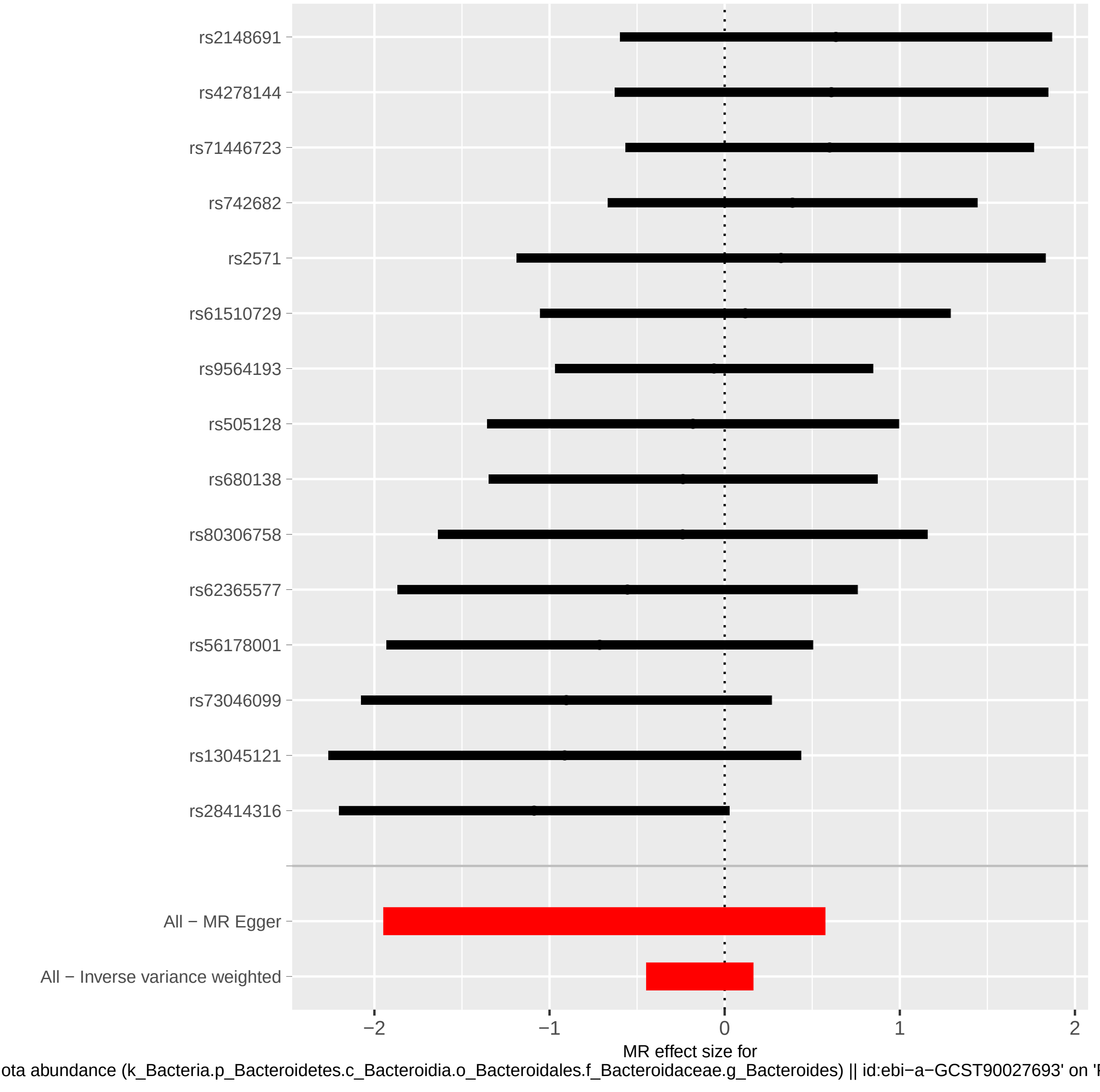

rs2148691
rs4278144
rs71446723
rs742682
rs2571
rs61510729
rs9564193
rs505128
rs680138
rs80306758
rs62365577
rs56178001
rs73046099
rs13045121
rs28414316
All − MR Egger
All − Inverse variance weighted
−2
−1
0
1
2
MR effect size for
ota abundance (k_Bacteria.p_Bacteroidetes.c_Bacteroidia.o_Bacteroidales.f_Bacteroidaceae.g_Bacteroides) || id:ebi−a−GCST90027693' on '
